# Le Fort I Osteotomy for Removal of an Extensive Complex Odontoma Involving the Maxillary Sinus: A Case Report and Literature Review

**DOI:** 10.1155/crid/3412161

**Published:** 2026-07-17

**Authors:** Yuri de Lima Medeiros, Lívia Marques dos Santos, Zarife Tirapani Adum Resende, Bruno Augusto Benevenuto de Andrade, Daniel Amaral Alves Marlière

**Affiliations:** ^1^ Department of Stomatology, School of Dentistry, São Paulo University, São Paulo, São Paulo, Brazil, usp.br; ^2^ University Hospital, Federal University of Juiz de Fora, Juiz de Fora, Minas Gerais, Brazil, ufjf.br; ^3^ Department of Pathology and Oral Diagnosis, School of Dentistry, Federal University of Rio de Janeiro, Rio de Janeiro, Rio de Janeiro, Brazil, ufrj.br; ^4^ Department of Clinical Dentistry, Dentistry School, Federal University of Juiz de Fora, Juiz de Fora, Minas Gerais, Brazil, ufjf.br

**Keywords:** maxillary sinus, odontoma, orthognathic surgery, osteotomy

## Abstract

Odontoma is a common odontogenic tumour in the maxillomandibular region, but its occurrence in the maxillary sinus is rare. Small‐ to medium‐sized odontomas can usually be removed without difficulties, depending on their location and proximity to adjacent structures. Nevertheless, access to extensive odontomas may be complicated. The aim of this study was to report a case of surgical removal of an extensive complex odontoma from within the maxillary sinus by using the Le Fort I osteotomy whilst also providing a review of the existing literature on intrasinus complex odontoma, with particular emphasis on surgical management. A 22‐year‐old male patient presented with an asymptomatic, well‐defined radiopaque lesion in the right maxillary sinus, measuring approximately 40 × 25 × 46 mm. The treatment consisted of surgical removal by using the Le Fort I osteotomy to facilitate access, full excision of the lesion and maintenance of bone integrity. Histological analysis revealed disorganised enamel matrix and dentinoid material with areas of calcification, consistent with the diagnosis of complex odontoma. The 1‐year follow‐up showed good healing, stable occlusion and no complications. Le Fort I osteotomy may be a safe and effective option for extensive intrasinus complex odontomas, particularly when posterior extension limits conventional access. However, its greater surgical complexity and potential morbidity require careful selection based on lesion size and posterior extension within the sinus.

## 1. Introduction

The term odontoma was first introduced in 1867 by Paul Broca to describe any tumour lesion of odontogenic origin [1]. The World Health Organisation [2] classifies it as a mixed benign odontogenic tumour. Odontoma is currently regarded not as a true neoplasm, but as a hamartomatous malformation composed of dental tissues, predominantly enamel and dentine, with varying amounts of pulp and cementum [3, 4]. Representing approximately 48.4% of all odontogenic tumours, this lesion is most commonly diagnosed during the first two decades of life [5]. It can be further classified as compound (with multiple tooth‐like structures) and complex (an amorphous mass of disorderly arranged dental tissues), in which the latter usually occurs in the posterior region of the mandible or anterior region of the maxilla, but is rare in the maxillary sinus [2, 6]. Although uncommon in the maxillary sinus, complex odontomas in this location may remain asymptomatic for long periods and reach considerable dimensions before diagnosis. Progressive enlargement may result in sinonasal symptoms, bone expansion, tooth displacement, orbital floor involvement and increased surgical complexity due to the proximity of vital anatomical structures [3, 4, 7, 8].

Management of odontomas consists of periodic follow‐up or surgical excision. Small‐ to medium‐sized odontomas can usually be removed without difficulty, depending on their location and proximity to adjacent structures. Nevertheless, access to extensive odontomas may be complicated [3, 6]. In 1988, Armstrong and Bhardwaj were the first to report the use of a Le Fort I osteotomy to access and remove an odontogenic tumour [7]. Although the technique is well established for managing lesions in the midface, its application for intrasinus complex odontomas remains exceptionally rare. To date, only two cases of maxillary sinus complex odontomas treated through a Le Fort I osteotomy have been documented in the literature [7, 8]. The present report therefore represents the third known instance of this surgical approach and, to our knowledge, the most extensive intrasinus complex odontoma ever managed using Le Fort I osteotomy. Accordingly, the present study describes the surgical management of this unusually large lesion and provides an updated review of similar cases.

## 2. Case Presentation

A 22‐year‐old male patient with no systemic disease initially sought dental care for orthodontic treatment. A panoramic radiograph incidentally revealed a radiopaque lesion in the right maxillary sinus, and the patient was subsequently referred to an Oral and Maxillofacial Surgery service. Clinically, the patient presented with no facial swelling or symptoms suggestive of pathology in the right maxillary sinus. The patient underwent image acquisition by means of cone beam computed tomography (CBCT) operating with a field of view (FOV) of 6 × 8 cm, an acquisition time of 17 s, 120 kVp, 5 mA, an isotropic voxel size of 0.4 mm and 14‐bit grey levels. The tomographic images were segmented and reconstructed three‐dimensionally, indicating the presence of a lesion in the sinus cavity in association with a retained tooth (i.e. second molar) and a hyperdense pattern suggestive of mineralised tissue. From multiplanar reconstructions of the tomographic images, it was observed that the lesion showed well‐defined margins extending anteroposteriorly and lateromedially for approximately 40 and 25 mm, respectively, and superoinferior extension of 46 mm (Figure 1). An incisional biopsy was performed under local anaesthesia through an intraoral vestibular approach in the right posterior maxilla. A mucoperiosteal flap was elevated, and a bony window measuring approximately 10 mm in diameter was created in the lateral wall of the maxillary sinus near the first molar region to access the lesion. Representative tissue samples were obtained using a trephine bur and a tapered fissure bur, allowing collection of both peripheral and deeper portions of the lesion. No intraoperative or postoperative complications, including wound dehiscence or sinus‐related symptoms, were observed.

**Figure 1:**
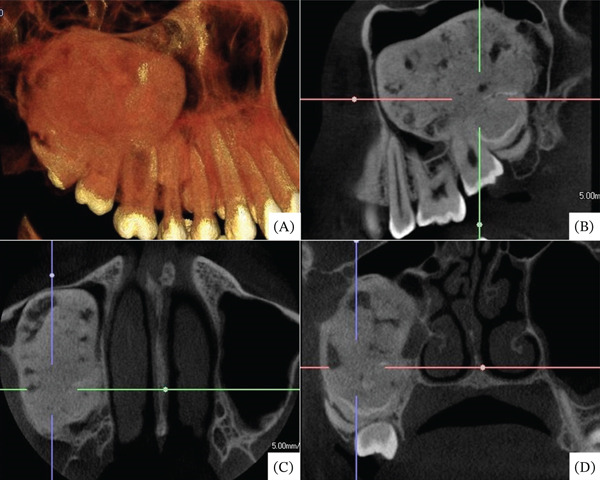
Preoperative CBCT evaluation of the right maxillary sinus. (A) Three‐dimensional reconstruction demonstrating a large, hyperdense mass occupying most of the right maxillary sinus. (B) Sagittal reconstruction showing a well‐defined, predominantly hyperdense lesion with internal structures suggestive of dental tissue. (C) Axial reconstruction illustrating the lesion′s lateromedial extension (approximately 25 mm) and its intimate relationship with the sinus walls. (D) Coronal reconstruction highlighting the anteroposterior (approximately 40 mm) and superoinferior (approximately 46 mm) extent of the lesion.

The surgical procedure for complete removal of the lesion was performed under general anaesthesia. Surgical access to the maxilla was obtained buccally by detachment of the subperiosteal nonkeratinised mucosa extending from the floor of the nasal fossa to the pterygomaxillary region. A Le Fort I osteotomy was then performed by using a reciprocating saw (Stryker CORE System) and completed with chisels. After osteotomy in the pterygomaxillary region and maxillary mobilisation (Figure 2A), the access to the sinus cavity was achieved. Because the lesion was closely associated with the maxillary second and third molars, both teeth were removed during the same surgical procedure. During its extraction, an oroantral communication occurred, and a combined vestibular and palatal sliding flap was immediately performed, allowing primary closure of the communication.

**Figure 2:**
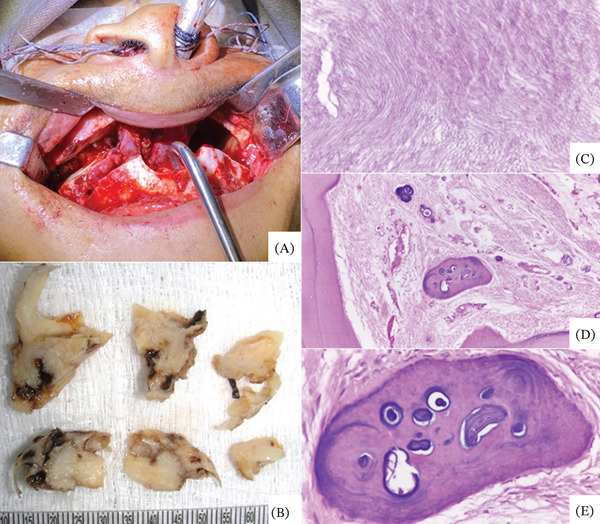
Intraoperative view, macroscopic specimens and histopathological findings. (A) Surgical access following Le Fort I osteotomy and maxillary down‐fracture, enabling full visualisation of the maxillary sinus. (B) Macroscopic view of the excised fragments, showing irregular contours, hard consistency and heterogeneous colour. (C–E) Histopathological sections demonstrating disorganised enamel matrix and dentinoid tissue with areas of calcification, confirming the diagnosis of complex odontoma (H&E, 100x).

The lesion was fragmented to permit complete removal whilst preserving the maxillary sinus walls. The maxillary walls were then levelled using rotary burs. An interocclusal surgical guide was positioned to stabilise the maxilla and mandible through maxillomandibular fixation. Repositioning of the maxilla was achieved with a 2.0 fixation system using four L‐shaped titanium plates (Engimplan, Materialise Company, Rio Claro, São Paulo, Brazil) placed in the piriform and zygomaticomaxillary regions bilaterally. The excised specimens (Figure 2B,C) exhibited an irregular shape, rough surface, hard consistency and brownish colour and were submitted for histopathological analysis. Microscopic examination revealed disorganised enamel matrix and dentinoid material with calcification areas, consistent with a complex odontoma (Figure 2D–F).

Postoperative CBCT imaging showed correct maxillary positioning and complete removal of the tumour (Figure 3). At the 1‐year follow‐up, the patient presented a favourable clinical evolution and stable occlusion. A transient episode of postoperative sinusitis related to haemosinus was successfully managed with antibiotic therapy. Infraorbital sensory function recovered uneventfully, and no persistent sinonasal symptoms were observed during follow‐up. No pulp vitality testing was performed, as the adjacent teeth were not directly involved by the lesion, whereas the second and third molars associated with the tumour were removed during surgery. A residual bone defect was noted in the alveolar process along the floor of the maxillary sinus (Figure 3B), corresponding to the area previously occupied by the removed second molar, showing a stable appearance and no fistula formation.

**Figure 3:**
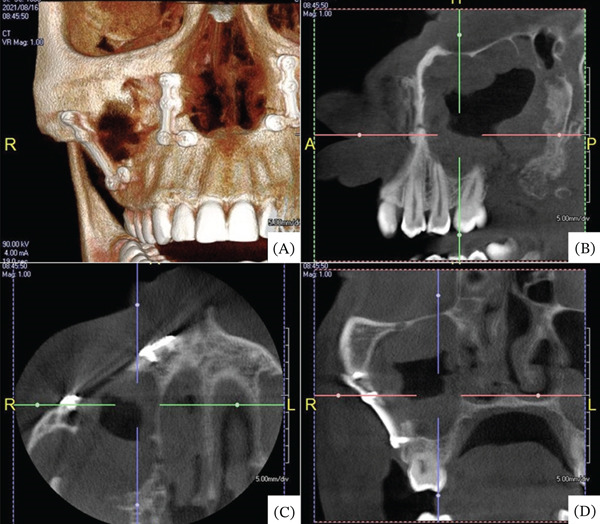
One‐year postoperative CBCT of the right maxillary sinus. (A) Three‐dimensional reconstruction showing stable repositioning of the maxilla with adequate fixation using titanium miniplates. (B) Sagittal reconstruction confirming complete excision of the lesion and demonstrating a residual bone defect in the alveolar process along the floor of the maxillary sinus, corresponding to the area previously occupied by the removed second molar. (C) Axial reconstruction showing restored sinus aeration, mild residual mucosal thickening in the right maxillary sinus, preservation of the sinus walls and no evident discontinuity of the lateral nasal wall. (D) Coronal reconstruction illustrating preserved anatomical contours and normal postoperative healing.

## 3. Discussion

To enable comparison with previously reported cases, a literature search was conducted in the PubMed database using the keywords ‘complex odontoma’ and ‘maxillary sinus’. A total of 20 cases published up to February 2026 were identified. One study was excluded due to insufficient description of the surgical approach [9]. Amongst the included reports, one article described two patients. The main characteristics of these cases are summarised in Table 1.

**Table 1:** Description of the cases of complex odontoma in the maxillary sinus published in the literature until 2026.

Authors (years)	Country	Gender and age	Side	Size	Clinical aspects and symptoms	Complications	Treatment	Follow‐up
Present study (2026)	Brazil	Male, 22	Right	40 × 46 mm	Asymptomatic	Sinusitis	Le Fort I osteotomy with fixation of 4 titanium miniplates	12 months
Agarwal et al. (2023) [10]	Republic of Korea	Male, 33	Right	22 × 38 mm	Asymptomatic	Mild rhinorrhea	Conventional surgical excision with intraoral access	Not reported
Suk et al. (2025) [11]	India	Male, 60	Right	Not reported	Facial asymmetry; erupted brown‐yellowish hard mass; inflamed gum; pain; purulent secretion	Oroantral fistula	Conventional surgical excision with intraoral access	12 months
Gupta and Das (2015) [12]	India	Female, 20	Left	60 × 40 mm	Erupted brown‐yellowish hard mass; inflamed gum and deep pocket; pain; purulent foetid secretion; unilateral nasal obstruction	Diplopia; sinusitis	Conventional surgical excision with intraoral access; antral tamponade and nasal antrostomy	Not reported
Visioli et al. (2015) [13]	Brazil	Male, 21	Left	Not reported	Teeth 27 and 28 missing; bone expansion; elimination of buccal sulcus	Absent	Conventional surgical excision with intraoral access	24 months
Murphy et al. (2014) [14]	Ireland	Male, 13	Right	Not reported	Facial asymmetry; erupted hard mass	Cellulitis	Conventional surgical excision with intraoral access and extraoral incision in the upper lip	36 months
Arunkumar et al. (2012) [15]	India	Male, 22	Left	40 × 35 mm	Teeth 26, 27 and 28 missing; erupted brown‐yellowish hard mass; inflamed gum; submandibular palpable lymph nodes; pain; oedema; foetid odour; nasal obstruction	Absent	Conventional surgical excision with intraoral access at 2 different moments due to dehiscence of the surgical wound	6 months
Isler et al. (2009) [16]	Turkey	Male, 33	Right	45 × 40 mm	Tooth 17 missing; bone expansion; pain	Absent	Conventional surgical excision with intraoral access with fixation of 2 titanium miniplates	3 months
Korpi et al. (2009) [8]	Finland	Male, 14	Right	30 × 30 mm	Tooth 17 missing; bone expansion	Absent	Le Fort I osteotomy with fixation of 4 preadapted titanium miniplates	18 months
Sales and Cavalcanti (2009) [17]	Brazil	Male, 31	Left	Not reported	Bone expansion; elimination of buccal sulcus	Presence of an associated dentigerous cyst	Conventional surgical excision with intraoral access	Not reported
Singer et al. (2007) [18]	USA	Female 23	Left	30 × 30 mm	Bone expansion; Teeth 27 and 28 missing	Elevation of the orbital floor without perforation	Conventional surgical excision with intraoral access	Not reported
Cabov et al. (2005) [19]	Croatia	Male, 23	Left	30 × 30 mm	Bone expansion	Absent	Conventional surgical excision with intraoral access and nasal antrostomy	12 months
Mupparapu et al. (2004) [20]	USA	Female, 16	Left	Not reported	Painless bone expansion; root resorption in Teeth 25 and 26; Tooth 27 missing	Oroantral fistula; elevation of the orbital floor without perforation	Conventional surgical excision with intraoral access	Not reported
Clayman and Marentette (1989) [21]	USA	Male, 28	Right	Not reported	Tooth 18 missing; nasal obstruction; otalgia; sinus congestion	Absent	Conventional surgical excision with intraoral access	Not reported
Armstrong and Bhardwaj (1988) [7]	Canada	Female, 18	Right	30 × 30 mm	Tooth 18 missing; mobility of Tooth 17 due to root resorption caused by the tumour; bone expansion	Absent	Le Fort I osteotomy with fixation of 6 transosseous wires	3 months
Shatz and Calderon (1987) [22]	Israel	Male, 29	Left	35 × 35 mm	Bone expansion; purulent exudate	Oroastral fistula; cellulitis	Conventional surgical excision with intraoral access	8 months
Visscher et al. (1982) [23]	Turkey	Female, 21	Left	30 × 30 mm	Tooth 27 missing; bone expansion; cervicoanterior palpable lymph node; pain	Absent	Conventional surgical excision with intraoral access	Not reported
		Male, 17	Left	50 × 30 mm	Teeth 27 and 28 missing; bone expansion	Presence of an associated dentigerous cyst	Conventional surgical excision with intraoral access	Not reported
Curreri et al. (1975) [24]	USA	Male, 12	Right	Not reported	Teeth 17 and 18 missing	Absent	Conventional surgical excision with intraoral access	Not reported
Caton et al. (1973) [25]	USA	Male, 48	Left	15 × 10 mm	Tooth 28 missing; nasal obstruction; persistent headache	Sinusitis	Conventional surgical excision with intraoral access	2 weeks
Christensen (1956) [26]	USA	Female, 11	Left	60 × 60 mm	Teeth 26, 27 and 28 missing; bone expansion	Diplopia; astigmatism	Conventional surgical excision with intraoral access	6 months

The mean age of the patients, including the present case, was 24.5 ± 11.9 years, ranging from 11 to 60 years. The male:female ratio was 2.5:1. Data demonstrated that intrasinus complex odontoma is more prevalent on the left side (57%), with a mean size of 36.9 × 34.6 mm. However, there is divergence in the literature as studies report that most of these lesions are detected during the second decade of life (mean age of 14 years), with little or no difference between genders [4].

Although intrasinus odontoma is not common, the maxillary sinus is a site susceptible to pathologies of odontogenic origin due to its close anatomical relationship with teeth and periodontal tissues [26]. As well as in the present case, an odontoma is usually found as an incidental radiographic finding, but the involvement of the maxillary sinus should be carefully evaluated, and the lesion should be removed because its growth can lead to local and systemic impairment. Amongst the possible complications associated with odontoma, one can cite deviation or absence of tooth eruption, alveolar bone expansion [7, 8, 13, 16, 18, 20], regional lymphadenopathy [15, 23], nasal obstruction [12, 15, 21, 25], epistaxis, diplopia and sinusitis [12, 26]. Moreover, cystic formation may concomitantly occur, resulting in bone destruction [17, 23].

Different surgical approaches have been proposed for the removal of lesions in the middle third of the face. The Caldwell–Luc technique was first described by George Caldwell in 1893 and Henri Luc in 1897 as a surgical approach for lesions in the maxillary sinus [27]. This approach provides direct access to the maxillary sinus through its anterior and lateral walls; however, its visualisation is limited, particularly for large or posteriorly located maxillary lesions. In such cases, surgical access becomes restricted, increasing the risk of incomplete excision and inadvertent injury to vital structures [8]. When extensive lesions are removed through Caldwell–Luc, permanent bony defects frequently remain in the sinus walls, and regeneration of these areas is often incomplete, potentially resulting in soft‐tissue collapse into the antral cavity [8]. More recently, endoscopic transnasal and combined endoscopic‐assisted approaches have also been described for the management of lesions involving the maxillary sinus. Elghobashy et al. [28] reported favourable outcomes using a combined endoscopic sinus surgery and endoscopic‐assisted Caldwell–Luc approach for an extensive maxillary sinus lesion, highlighting improved visualisation and reduced cosmetic morbidity. Similarly, Şimşek and Üstündağ [29] described the use of endoscopic assistance to facilitate the complete removal of odontogenic structures located beyond the direct surgical field within the maxillary sinus. Nevertheless, in extensive lesions with marked posterior extension and substantial mineralised content, Le Fort I osteotomy may still provide broader surgical exposure and greater operative control.

As an alternative to the conventional surgical approaches, the Le Fort I osteotomy provides better access to lesions located in the posterior region of the maxilla, enabling direct visualisation of the entire maxillary sinus, nasal cavity, infraorbital region, posterior maxilla and pterygomaxillary junction [30]. This panoramic visualisation facilitates precise delineation of osteotomy margins, safer dissection around vital structures and predictable removal of lesions that extend deeply or posteriorly within the sinus [8]. The horizontal osteotomy line can also be adjusted to accommodate lesions approaching the orbital floor or skull base, further enhancing surgical control. In addition to avoiding external scars, the Le Fort I approach allows conservative bone removal and minimises postoperative defects of the sinus walls [30].

Importantly, evidence suggests that this technique may also contribute to lower recurrence rates in aggressive lesions. Alstad and Abtahi [31], in a series of nine keratocystic odontogenic tumours of the maxillary sinus, demonstrated that the expanded access provided by Le Fort I osteotomy facilitated complete removal and was associated with favourable long‐term outcomes. Furthermore, this approach is highly versatile and can be combined with reconstructive procedures, endosseous implant placement or even concomitant correction of facial deformities within the same surgical access when required [32].

In the present case, the lesion extended posteriorly towards the pterygomaxillary region and superiorly close to the orbital floor, occupying a substantial portion of the sinus cavity. Although endoscopic‐assisted approaches have recently been described for selected maxillary sinus lesions [28, 29], the extensive mineralised nature and anatomical extent of the tumour could have limited manoeuvrability and controlled fragmentation through a more restricted access. Therefore, Le Fort I osteotomy was considered more suitable to facilitate direct visualisation and controlled removal [8]. In addition to the present study, only Armstrong and Bhardwaj [7] and Korpi et al. [8] reported the use of Le Fort I osteotomy for the removal of an intrasinus complex odontoma. In all reports, the lesions presented considerable size and posterior extension within the maxillary sinus, making conventional intraoral access potentially limited. However, some relevant differences can be observed amongst these cases. Armstrong and Bhardwaj [7] reported the use of transosseous wire fixation after maxillary down‐fracture, whereas both Korpi et al. [8] and the present study employed rigid fixation with titanium miniplates, allowing stable repositioning of the maxilla and favourable postoperative recovery. Compared with the previously published cases, the present lesion showed greater superoinferior extension and occupied a substantial portion of the sinus cavity, increasing the technical difficulty of surgical access and reinforcing the importance of broad visualisation during tumour removal. Furthermore, unlike several cases managed through conventional approaches that reported complications such as persistent oroantral fistula, wound dehiscence or need for secondary procedures [12, 15, 20, 22], the present case showed favourable postoperative evolution, with only transient sinusitis and a buccosinusal communication, both successfully managed without long‐term sequelae.

This technique offers predictable surgical control, preserves occlusion and is associated with a low rate of postoperative morbidity. The possible complications are the same ones which may occur during the surgical correction of a dentofacial deformity. Haas Junior et al. [33] performed a systematic review and found that only 8.5% of the patients submitted to Le Fort I osteotomy had a postoperative complication. The most frequent adverse effects include haemorrhage, pulp necrosis, bucosinusal fistulas (oroantral), lesions of the nasolacrimal duct and secondary infections [30, 33]. Soares et al. [30] reported the use of a Le Fort I osteotomy for the removal of a large ossifying fibroma measuring 38.9 × 35.4 mm, in which the only postoperative event was a mild oroantral communication on Day 15, attributed to a small mucosal perforation during lesion detachment and successfully managed with a vestibular flap. In the present case, there were no immediate or long‐term complications attributable to the Le Fort I approach. The residual bone defect seen postoperatively corresponded to the area previously occupied by the maxillary second molar, which required extraction due to its association with the tumour. This defect reflects bone loss inherent to the tooth removal rather than a consequence of the osteotomy. The transient buccosinusal communication created during extraction was repaired immediately, and the patient healed without fistula or sinus‐related symptoms at the 1‐year follow‐up.

In conclusion, Le Fort I osteotomy was considered safe and effective for the removal of an extensive intrasinus complex odontoma. However, given its greater surgical complexity, potential morbidity and associated burden, its indication should be based on lesion size and posterior extension within the sinus, particularly when conventional approaches would provide limited access. In selected cases, it enables improved visualisation, complete tumour removal and preservation of maxillary bone integrity, supporting favourable outcomes.

## Author Contributions

Yuri de Lima Medeiros: formal analysis, data curation, visualisation, writing—original draft and writing—review and editing; Lívia Marques dos Santos: investigation, data curation, resources and writing—review and editing; Zarife Tirapani Adum Resende: investigation, data curation, resources and writing—review and editing; Bruno Augusto Benevenuto de Andrade: investigation, resources, validation and writing—review and editing; Daniel Amaral Alves Marlière: conceptualisation, methodology, investigation, project administration, supervision, validation and writing—review and editing.

## Funding

No funding was received for this manuscript.

## Disclosure

All authors have read and approved the final version of the manuscript. Daniel Amaral Alves Marlière had full access to all of the data in this study and takes complete responsibility for the integrity of the data and the accuracy of the data analysis. A preprint has previously been published [34].

## Ethics Statement

All procedures performed in studies involving human participants were in accordance with the ethical standards of the institutional and national research committee and with the 1964 Helsinki Declaration and its later amendments or comparable ethical standards.

## Consent

Informed consent was obtained from the patient. Furthermore, the participant has consented to the submission of the case report to the journal.

## Conflicts of Interest

The authors declare no conflicts of interest.

## Data Availability

The data that support the findings of this study are available from the corresponding author upon reasonable request.
